# The h-index is no longer an effective correlate of scientific reputation

**DOI:** 10.1371/journal.pone.0253397

**Published:** 2021-06-28

**Authors:** Vladlen Koltun, David Hafner

**Affiliations:** 1 Intelligent Systems Lab, Intel, Jackson, WY, United States of America; 2 Intelligent Systems Lab, Intel, Neubiberg, Germany; Universitat de Barcelona, SPAIN

## Abstract

The impact of individual scientists is commonly quantified using citation-based measures. The most common such measure is the h-index. A scientist’s h-index affects hiring, promotion, and funding decisions, and thus shapes the progress of science. Here we report a large-scale study of scientometric measures, analyzing millions of articles and hundreds of millions of citations across four scientific fields and two data platforms. We find that the correlation of the h-index with awards that indicate recognition by the scientific community has substantially declined. These trends are associated with changing authorship patterns. We show that these declines can be mitigated by fractional allocation of citations among authors, which has been discussed in the literature but not implemented at scale. We find that a fractional analogue of the h-index outperforms other measures as a correlate and predictor of scientific awards. Our results suggest that the use of the h-index in ranking scientists should be reconsidered, and that fractional allocation measures such as h-frac provide more robust alternatives.

## Introduction

The h-index, proposed by Hirsch in 2005 [1], has become the leading measure for quantifying the impact of a scientist’s published work. The h-index is prominently featured in citation databases such as Google Scholar, Scopus, and Web of Science. It informs hiring, promotion, and funding decisions [2–4]. It thereby shapes the evolution of the scientific community and the progress of science.

Numerous variants of the h-index have been explored, and sophisticated alternatives have been proposed [5, 6]. None of these has displaced the h-index as the dominant measure of a scientist’s output. The endurance of the h-index can be attributed to a number of characteristics. First, it summarizes a scientist’s output in a single number that can be readily used for comparison and ranking. Second, it does not require a minimal number of publications or career length, and can thus be computed for scientists at all career stages. Third, it does not require tuning thresholds or parameters. Fourth, it is easily interpretable. Lastly, criticism notwithstanding, the h-index is seen as a robust measure of an individual scientist’s impact [7–10].

Science continues to evolve and publication patterns change over time [11]. Here we report an extensive empirical evaluation of individual research metrics. Since publication patterns differ across scientific fields [12–14], we collect large datasets in four fields of research: biology, computer science, economics, and physics. In each field, we consider 1,000 most highly cited researchers and trace their published output and its impact through two bibliographic data platforms: Scopus [15] and Google Scholar [16]. The resulting datasets comprise 1.3 million articles and 102 million citations identified via Scopus and 2.6 million articles and 221 million citations identified via Google Scholar (S3 Fig).

We have cross-referenced the scientists in our datasets against lists of recipients of scientific awards that indicate recognition by the scientific community: Nobel Prizes, Breakthrough Prizes, membership in the National Academies, fellowship of the American Physical Society, Turing Award, fellowship of the Econometric Society, and other distinctions (S4 Fig and S1 Table). Among the 4,000 authors in our dataset, 75.6% have no such awards, 13.3% have one award, 5.1% have two, and 6.0% have three or more (S4D Fig). Our basic methodology is to correlate rankings induced by scientometric measures with rankings induced by scientific awards. The assumption is that a citation-based measure that more reliably uncovers laureates of elite awards is a more veridical indicator of scientific reputation [6, 17]. Since publication, citation, and award patterns differ substantially across fields, we conduct parallel experiments in the four fields of research. To confirm the robustness of the findings, the studies are replicated across the two bibliographic platforms (Scopus and Google Scholar).

A number of prior studies are related to our work. Sinatra et al. [6] analyze the careers of 2,887 physicists in the APS dataset and 7,630 scientists in the Web of Science database, considering approximately one million publications in total. Their study includes evaluations that correlate individual scientific impact indicators with scientific awards. However, this is performed on a limited scale, taking into account only Nobel prizes in physics and Dirac and Boltzmann medals as indicators of scientific reputation. Considering publication and citation data of 84,116 scientists, Ioannidis et al. [17] investigate a number of citation indicators based on how well they capture Nobel prize winners from the years 2011–2015. The recent study of Ayaz and Masood [18] evaluates indices of researchers’ impact by analyzing 236,416 publications in the area of computer science. Their comparison of bibliometric indices is based on 47 award winners in their dataset.

Our study is conducted on a much larger scale. We analyze millions of articles in four different research fields that are cited hundreds of millions of times. We collect more than 10,000 awards and trace 1,848 distinct awards to the 4,000 scientists in our dataset. (See S1 Text.) Most importantly, our datasets have yearly temporal granularity from 1970 onwards. This enables detailed evaluation of the temporal evolution of the effectiveness and predictive power of research metrics that, to the best of our knowledge, has not been presented before.

Our first major finding is that the effectiveness of scientometric measures is declining. For example, the correlation of the h-index with scientific awards in physics has dropped from 0.34 in 2010 to 0.00 in 2019 (Kendall’s *τ*, Scopus physics dataset). This is associated with changing authorship patterns, including a higher prevalence of hyperauthorship. Our second major finding is that fractional allocation of citations among coauthors can mitigate this decline [14, 19, 20]. In particular, for each measure we study, its fractional counterpart is a better correlate and predictor of scientific awards. Among all measures, a fractional analogue of the h-index, h-frac, consistently outperforms alternatives.

We test the robustness of the findings via controlled experiments across datasets. The main findings hold in all conditions: fractional allocation improves the effectiveness and predictive power of research metrics, and h-frac is consistently the most reliable bibliometric indicator. Our results suggest that the use of the h-index in ranking scientists should be reconsidered, and that fractional allocation measures such as h-frac provide more robust alternatives. The data also indicate, contrary to concerns expressed in the literature, that fractional allocation measures are not antithetic to collaboration. Our findings can lead to more effective distribution of resources and thus accelerate scientific discovery [21]. Our data, methodology, and findings may also have broader applications in the empirical analysis of science [11].

## Results

### Declining effectiveness of individual research metrics

**Fig 1:**
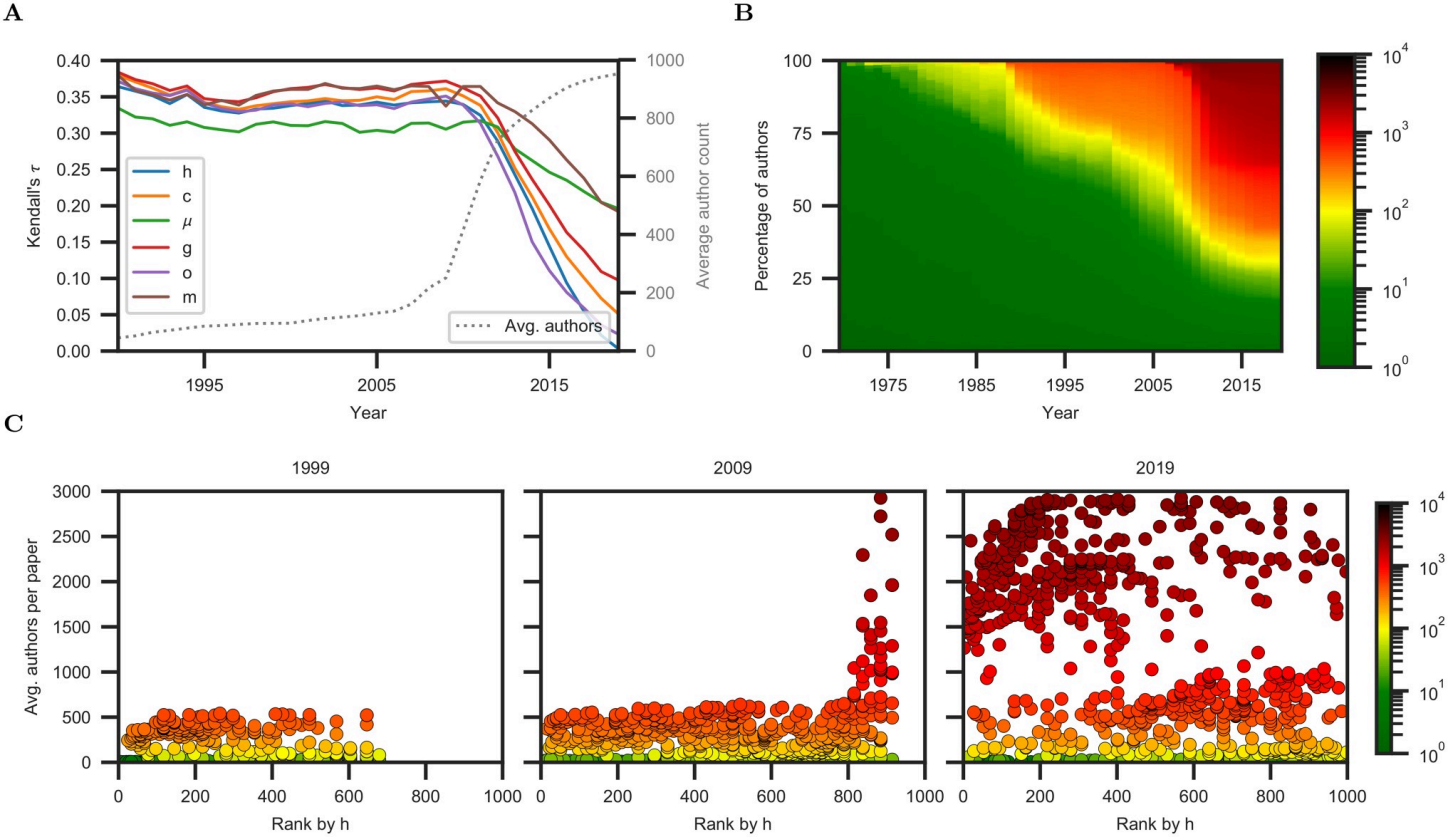
The effectiveness of scientometric measures is declining. (**A**) Effectiveness of scientometric measures as correlates of scientific awards in the Scopus physics dataset. (**B**) Color-coded distribution of the average number of coauthors per publication in this dataset. (**C**) Ranking of physicists by the h-index. Each data point is a scientist. Color and the vertical axis represent the average number of coauthors per publication.

Fig 1A shows the effectiveness of scientometric measures over the past 30 years. The effectiveness of a scientometric measure is quantified by the correlation between the ranking induced by this measure and the ranking induced by scientific community awards at a given point in time. Here we report Kendall’s *τ* on the Scopus physics dataset (see S6 Fig for other correlation criteria and datasets). In addition to the h-index (h), we evaluate the total number of citations to a scientist’s work (c), the mean number of citations per paper (*μ*, advocated by Lehmann et al. [22]), Egghe’s g-index [23], the o-index [24], and the median number of citations received by a scientist’s highly-cited papers (m, highlighted by Bornmann et al. [25]). (See S1 Text.)

As Fig 1A demonstrates, the effectiveness of scientometric measures has declined. The decline is particularly pronounced for the h-index. The effectiveness of the h-index, as measured by Kendall’s *τ*, varied between 0.33 and 0.36 from 1990 to 2010, but dropped to 0.00 by 2019 on the Scopus physics dataset. This is concomitant with a dramatic shift in authorship patterns, illustrated by the average number of coauthors per paper for highly-cited physicists. While the mean number of coauthors per publication, averaged across highly cited physicists, was 78 in 1994 and 121 in 2004, it rose to 952 in 2019, with 10% of the scientists having more than 2,441 coauthors per publication on average. (See S2 Dataset.)

This is further illustrated in Fig 1B, which shows the distribution of the average number of coauthors per paper for highly-cited physicists in each year from 1970 onwards. While small authorship teams were nearly universal in the beginning of this period (84% of the scientists had <10 coauthors per publication on average in 1980), the set of highly-cited physicists has come to be dominated by “hyper-collaborators”: 68% of the scientists had >100 coauthors per publication on average in 2019. Large-scale collaboration has been a feature of science for centuries, but joint authorship has been institutionalized on a new scale in the past decade [17]. Scientific consortia comprise thousands of authors who jointly author hundreds of publications [26]. All members of the consortium are listed as authors on all papers [27]. This has been referred to as hyperauthorship [28, 29]. Our results indicate that this behavior is reducing the effectiveness of established scientometric indicators. This is further illustrated in Fig 1C, which shows the ranking of physicists by h-index in 1999, 2009, and 2019. The hyper-collaborators have permeated the ranking.

### Fractional allocation

Are there scientific impact metrics that share the advantages of the h-index and are robust to contemporary publication patterns? Hirsch proposed a bibliometric indicator that takes authorship into account [30], but his mechanism requires recursive computation across the citation network and, even in its more tractable approximate form, is “particularly unkind to junior researchers” [30]. An alternative that inherits the simplicity of the h-index is to allocate citations fractionally among authors.

Derek de Solla Price [19] advocated distributing credit for a scientific publication among all authors to preclude undesirable publication practices: “The payoff in brownie points of publications or citations must be divided among all authors listed on the byline, and in the absence of evidence to the contrary it must be divided equally among them. […] If this is strictly enforced it can act perhaps as a deterrent to the otherwise pernicious practice of coining false brownie points by awarding each author full credit for the whole thing.” [19]. Since the introduction and broad adoption of the h-index [1], many variants and related measures have been proposed [5, 14, 31]. Some of these implement fractional allocation. Batista et al. [32] present a normalization of the h-index by the average number of authors of papers in the h-core. Wan et al. [33] perform a similar normalization, but use the square root of the average authors of papers in the h-core. Chai et al. [34] describe a variant of the h-index that is based on citation counts normalized by the square root of the number of authors per paper. Egghe [20] introduces alternative versions of the h- and g-index (see S1 Text) that use citation counts normalized by the number of authors. Egghe’s version of the h-index corresponds to the h-frac measure that we find to be particularly effective in our experiments. Note that the work of Egghe is purely theoretical and does not include any experiments with real bibliographic data [20]. Schreiber [35, 36] presents an alternative fractional allocation measure. Instead of using normalized citation counts, Schreiber proposes to first compute alternative (“effective”) publication ranks that are divided by the number of authors. These effective ranks are then used to determine the h_m_-index, akin to computing the h-index with unmodified publications ranks. A related alternative has also been proposed for the g-index [37, 38]. Other variants that apply different fractional allocation schemes can also be found in the literature [39–42]. While there exist bibliometric tools that implement fractional versions of the h-index [43, 44], we are not aware of published systematic empirical evaluation of fractional allocation measures with real bibliographic data, on a large scale (millions of articles), and across multiple scientific fields and data platforms. We contribute such an evaluation. Among other measures, we experimentally evaluate h-frac alongside the scientometric measures of Batista et al. [32] (h_I_), Schreiber [35, 36] (h_m_), Wan et al. [33] (h_p_), and Chai et al. [34] (h_ap_).

**Fig 2:**
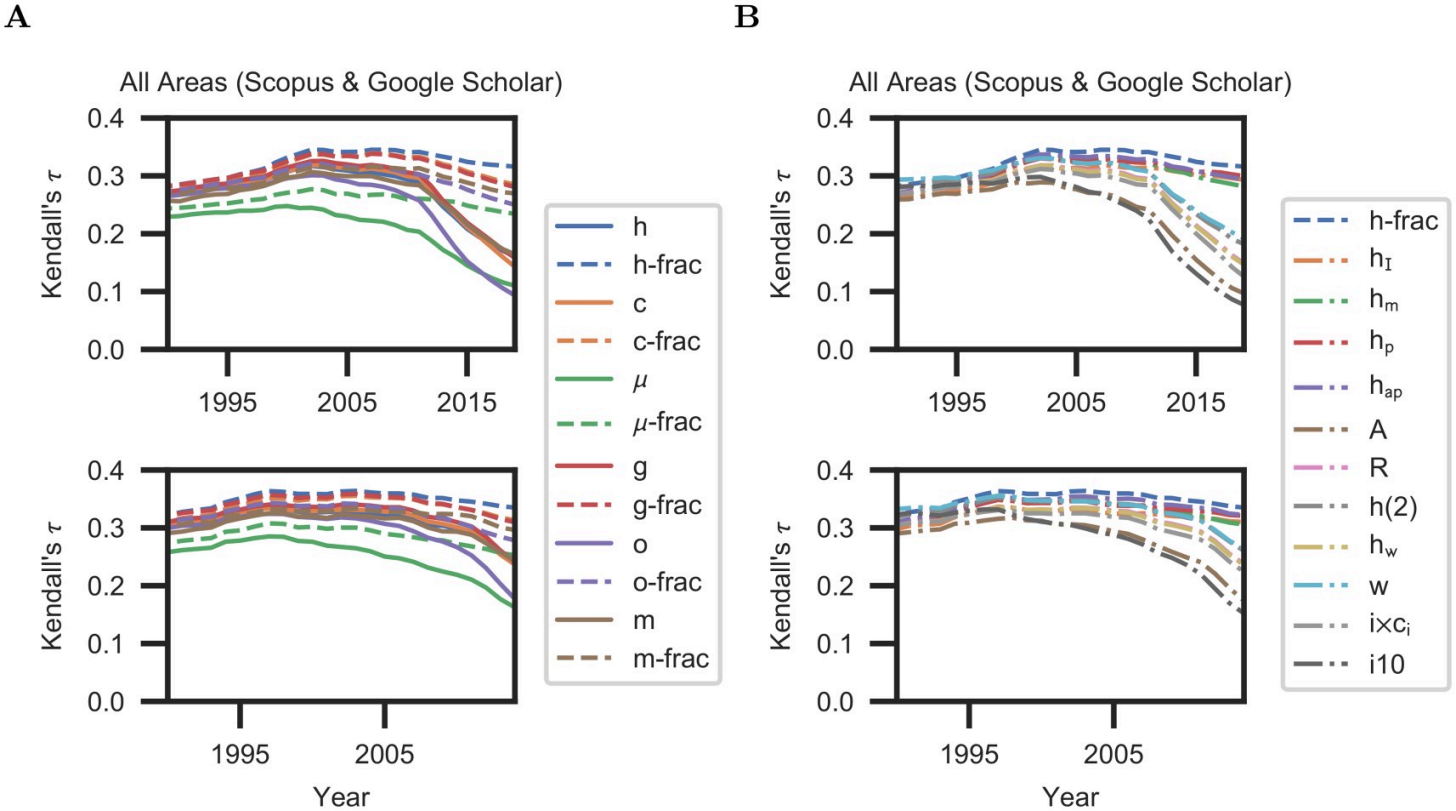
Effectiveness and predictive power of scientometric measures. In each subfigure, the top row depicts the correlation of bibliometric indicators and scientific awards, and the bottom row shows the predictive power five years into the future. (**A**) Evaluation across all research areas and data platforms (Scopus and Google Scholar). (**B**) Evaluation of h-frac alongside additional measures across all research areas and data platforms.

Fig 2A(top) contrasts the effectiveness of fractional allocation measures and traditional ones across all research fields and data platforms. We again measure the correlation of rankings induced by different bibliometric measures and scientific reputation as evidenced by awards bestowed by the scientific community. Detailed results for the individual research areas can be found in S6 Fig(left).

We find that fractional measures are significantly more effective correlates of scientific awards than unnormalized indicators such as the h-index. The fractional analogue of the h-index, h-frac, is the most effective measure across datasets (average *τ* = 0.32 in 2019, compared to 0.16 for the h-index; see S2 Table(top)). The effectiveness of fractional allocation measures is more stable over time than the effectiveness of their traditional counterparts. (For h-frac, average *τ* = 0.28 in 1989 and 0.32 in 2019; for the h-index, average *τ* = 0.27 in 1989 and 0.16 in 2019.)

### Predictive power and other measures

Next we evaluate the predictive power of different bibliometric measures. Prior studies have largely focused on the ability of measures to predict their own future values, or those of other bibliometric indicators [7, 10, 45]. In contrast, we study the ability of an indicator to predict a scientist’s future reputation as evidenced by scientific awards. (Hirsch recognized this as a meaningful goal when he wrote “how likely is each candidate to become a member of the National Academy of Sciences 20 years down the line?”, but did not operationalize this [7].) We measure the correlation of rankings induced by scientometric indicators in a given year (e.g. 2010) with rankings induced by awards in a future year (e.g. 2015). Higher correlation implies stronger ability to predict future scientific reputation based on present-day bibliometric data.

Fig 2A(bottom) reports predictive power five years into the future. The results are summarized across all research fields and data sources. The predictive power of the h-index has declined since its introduction (average *τ* = 0.32 in 2004 versus 0.24 in 2014). Other traditional indicators have also declined in effectiveness. Fractional measures are more predictive. h-frac has the highest predictive power across datasets and its predictive power is stable over time (average *τ* is 0.34 in 1994, 0.36 in 2004, and 0.33 in 2014).

We further evaluate h-frac alongside an extensive list of other scientometric measures [5, 16, 32–36, 46–51]. The results are summarized in Fig 2B. Measures that integrate some form of normalization by the number of coauthors (h-frac, h_I_, h_m_, h_p_, h_ap_) outperform measures that do not apply such normalization. h-frac is the best-performing measure in terms of both correlation with scientific awards and predictive power.

### Robustness of the findings

We now test the robustness of the findings in a number of additional controlled experiments.

**Fig 3:**
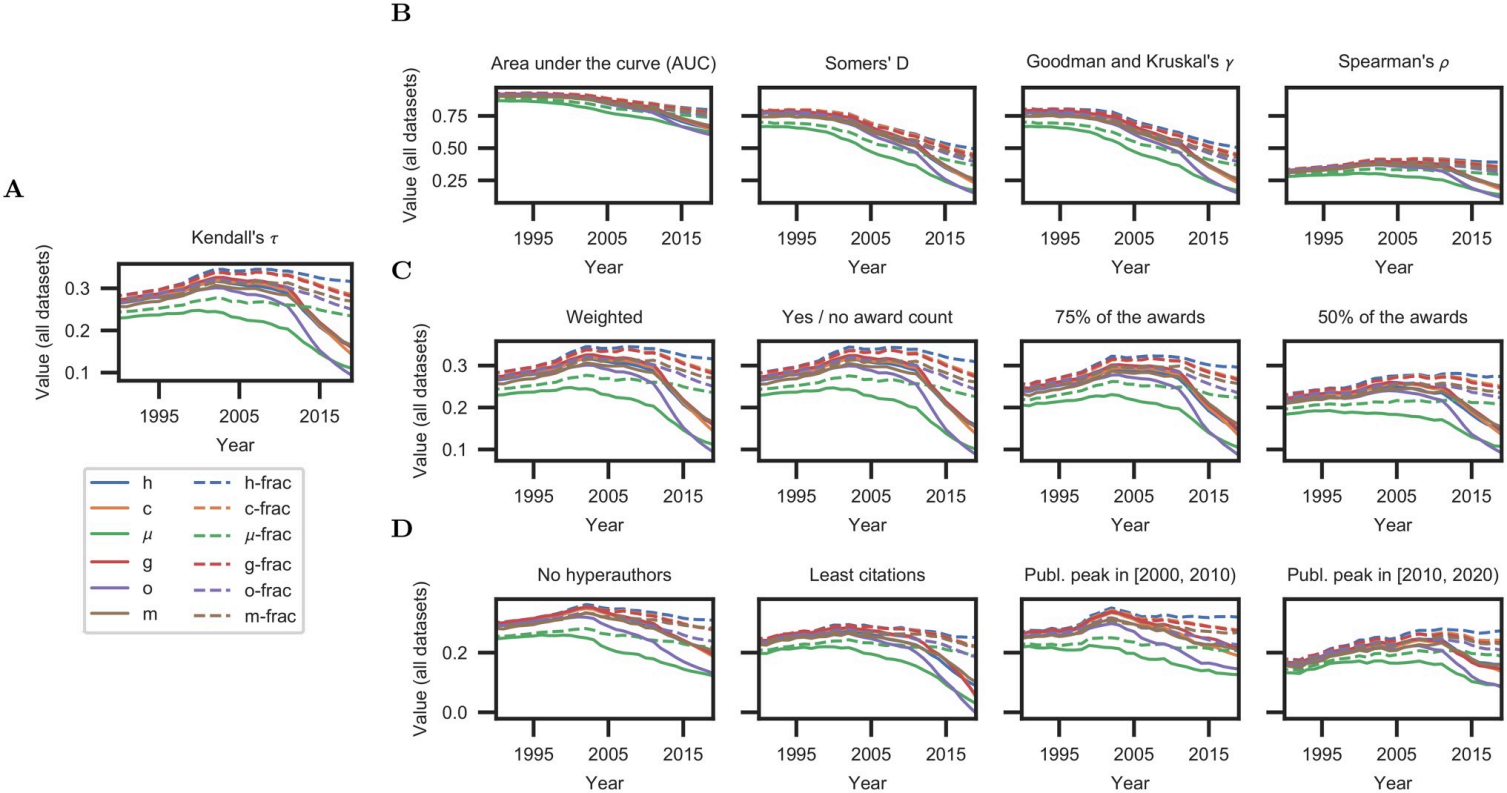
Controlled experiments that test the robustness of the findings. (**A**) Reference result from the main experiments (cf. Fig 2A(top)). (**B**) Corresponding results with other correlation statistics. (**C** and **D**) Results in different conditions: using subsets of awards, researchers, and different mechanisms for counting awards.

First, we repeat the experiments with different correlation statistics (see S1 Text). The results are summarized in Fig 3B, and detailed results for all research areas and data platforms can be found in S1 Text and S6 Fig. Fractional measures continue to outperform their traditional counterparts, and h-frac is the most reliable indicator.

Next we analyze robustness with respect to the set of scientific awards considered in our datasets. Our main experiments treated all awards equally, and ranked scientists by the total number of awards received. For example, a Nobel prize was given the same weight as membership in the National Academy of Sciences, and a scientist with two awards was ranked higher than a scientist with one award. To examine whether our findings are sensitive to this choice, we repeat the experiments under different conditions. First, we assign 10 times higher weight to awards with 100 or fewer laureates. (See S1 Table.) Second, we evaluate a design in which the number of awards does not affect a scientist’s ranking: a scientist with an award of any kind is ranked higher than a scientist with no awards, but all scientists with one or more awards are ranked equally. The results are summarized in Fig 3C(left) and presented in detail in S1 Text and S7 Fig. Our findings hold for both conditions. (The results remain consistent for other weighting factors and thresholds as well.)

To further assess sensitivity, we repeat the experiments with random subsets of awards (using 75% and 50% of awards in our database). The results are reported in Fig 3C(right) and S7 Fig. Our findings again hold. This demonstrates the robustness of our findings with respect to the considered awards and the matching procedure. (See S1 Text.)

Is the decline in the effectiveness of the h-index and other traditional scientometric measures solely due to the rise of hyperauthorship? To investigate this hypothesis, we curtail the effect of hyperauthorship by reproducing the experiments with the set of authors who have at most 100 coauthors per paper on average. The results in Fig 3D(left) show that our findings hold in this condition as well: we see a strong decline in the effectiveness of traditional measures, in contrast to the stable performance of their fractional counterparts. Hyperauthors appear to be an extreme manifestation of a broader shift in publication patterns. Hyperauthors themselves are not the main cause of the decline in the effectiveness of the h-index and other measures, and pruning hyperauthors from datasets does not avert this decline.

Next we perform experiments with different subsets of researchers. First we remove the most highly-cited researchers in our datasets and repeat the experiments with the bottom 50% of researchers in each field by number of citations. This examines whether our findings hold for researchers that are not at the very top of their fields in terms of citations. Then we analyze the effect of the main time period of a scientist’s work. (Details on the temporal coverage of the authors in our dataset can be found in S3 Fig.) To this end, we choose subsets of researchers that are active at different periods of time. Specifically, we test the subset of researchers whose peak productivity (in terms of number of publications) occurs during the years [2000, 2010), and another subset whose peak productivity occurs during the years [2010, 2020).

The results are summarized in Fig 3D and given in detail in S8 Fig. Our main findings are robust to all these perturbations and hold in all conditions: fractional allocation measures always outperform their traditional counterparts, and h-frac is the most reliable bibliometric indicator across all conditions.

### Correlation between scientometric measures

**Fig 4:**
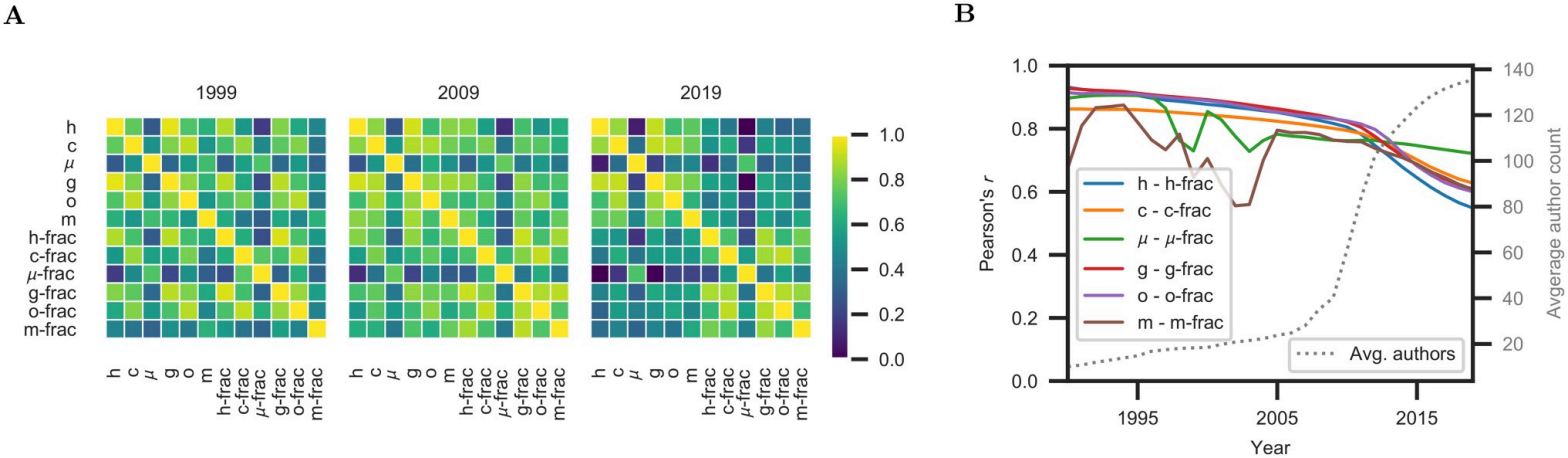
Correlation between scientometric measures. (**A**) Correlation matrices of scientometric measures in the years 1999, 2009, 2019. (**B**) Temporal evolution of correlations between traditional measures and their fractional counterparts.

Our experiments indicate that fractional allocation measures are superior to their traditional counterparts. To analyze this further, we investigate the correlation between different scientometric measures [17, 52]. To this end, we compute the correlation between each pair of measures, aggregated over all datasets (Fig 4A). To interpret the results, we consider three different 6x6 blocks in the correlation matrices:

(i) The *lower right* block summarizes the correlations between the fractional measures. It is quite stable over the years. All fractional measures are moderately correlated, with the exception of *μ*-frac. The lower correlation of *μ*-frac with the other fractional measures can be explained by the explicit normalization by the number of publications in *μ*-frac, which is absent in the other measures. As can be seen in the preceding results, *μ*-frac is the worst-performing measure among the fractional ones.
(ii) The *upper left* block summarizes the correlations between the traditional measures. These correlations are stable over time. The traditional measures are moderately correlated with each other, again with the exception of *μ*. This can again be attributed to the explicit normalization by the number of publications in *μ*.
(iii) The *lower left* block captures the correlations between the traditional and fractional measures. Notably, we observe that these correlations decrease significantly from 2009 to 2019. All correlation values decrease, including the correlations between the traditional measures and their direct fractional counterparts (the diagonal in the lower-left block). The measures *μ* and *μ*-frac stand out again, which can be attributed to the same factors as in the other blocks.

Why have the traditional and fractional measures become less correlated over time? We examine the temporal evolution of correlations between traditional measures and their fractional counterparts at finer granularity (Fig 4B). We see that the correlation decreases over time, with accelerated decline after 2010. Concurrently, the average number of authors per publication rises significantly. The two trends are strongly correlated. (E.g., the correlation between the correlation of h and h-frac and the average author count is −0.97.) Since accounting for the number of authors per publication is the central feature that distinguishes fractional measures from their traditional counterparts, we attribute the diminishing correlation between the measures to the changing publication culture, as reflected in the dramatic increase in the average number of authors per paper.

### Further analysis

**Fig 5:**
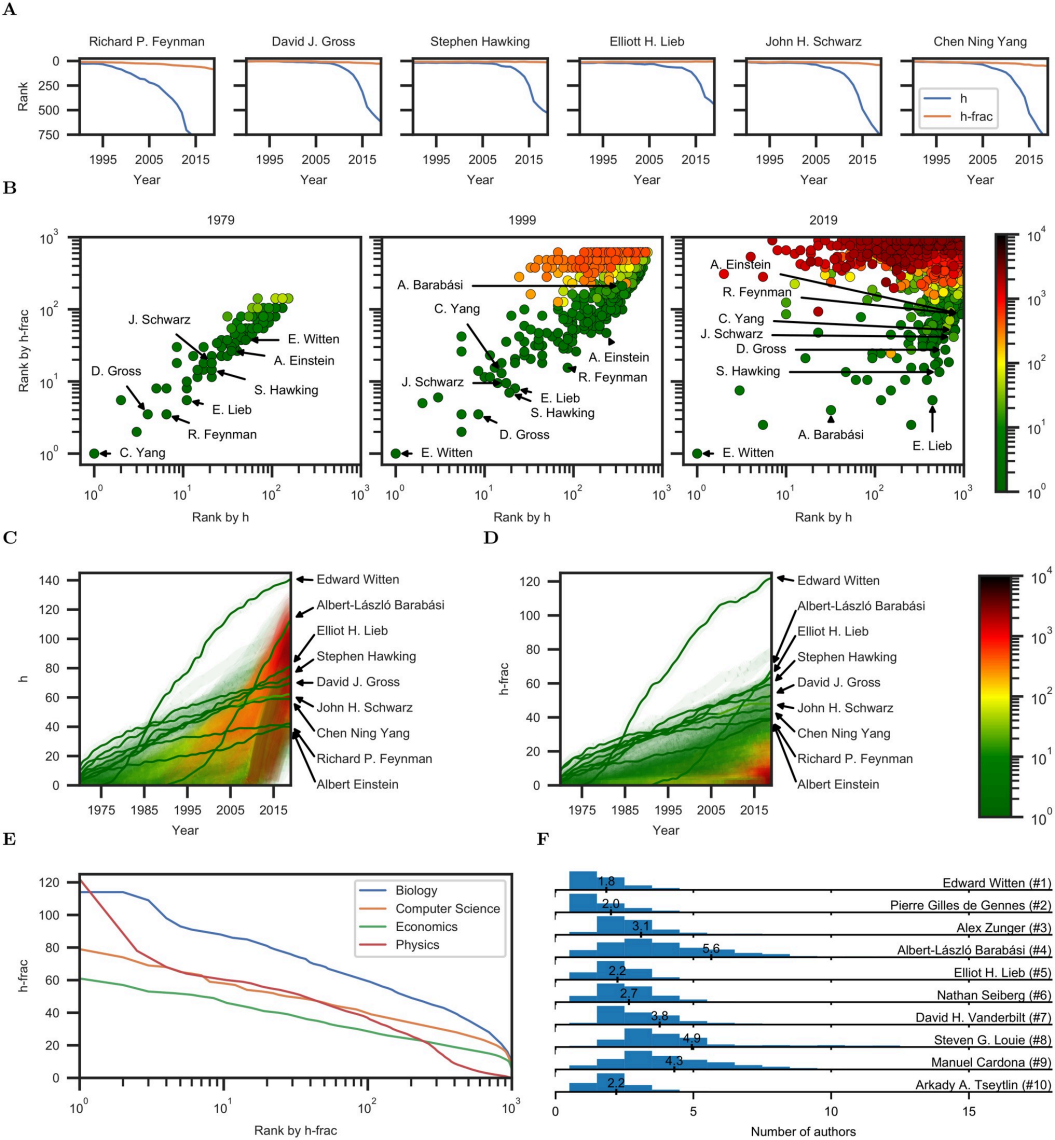
Further analysis. (**A**) Ranking induced by h and h-frac for a number of scientists in the Scopus physics dataset. (**B**) Comparison of rankings induced by h and h-frac in the Scopus physics dataset. Scientists are color-coded by the average number of coauthors per publication. (**C**) Evolution of the h-index of each scientist in the Scopus physics dataset over time. Each scientist is a curve. Color represents the average number of coauthors per publication. (**D**) Evolution of h-frac over time. (**E**) Distribution of h-frac values in each field of research. (**F**) Distribution of the number of authors per publication for 10 physicists with the highest h-frac in 2019.

Fig 5A provides a number of case studies that highlight the stability of h-frac and the deterioration of the h-index over time. These case studies are further illustrated in Fig 5B. The evolution of h and h-frac values over time is visualized in Fig 5C and 5D. Hyperauthors (red) acquire increasingly high h-indices over time, commonly rising above 80 by 2019. In contrast, their h-frac values remain low, predominantly less than 20. Fig 5E visualizes the distribution of h-frac values in the four fields of research. The top 100 scientists have h-frac values of 59 and higher in biology, 39 and higher in computer science, 37 and higher in physics, and 29 and higher in economics.

Fig 5F examines in detail the output of the 10 physicists with the highest h-frac in 2019. The data suggests that the h-frac measure is not antithetical to collaboration, which is associated with scientific progress [53–55]. Among physicists with the highest h-frac are prolific collaborators such as Albert-László Barabási (#4, 5.6 authors per publication on average), Steven G. Louie (#8, 4.9 authors per publication on average), and Manuel Cardona (#9, 4.3 authors per publication on average).

## Discussion

We have conducted a large-scale systematic analysis of scientometric measures. We have demonstrated that commonly used measures of a scientist’s impact have become less effective as correlates and predictors of scientific reputation as evidenced by scientific awards. The decline in the effectiveness of these measures is associated with changing authorship patterns in the scientific community, including the rise of hyperauthorship. We have also demonstrated that fractional allocation of citations among coauthors improves the robustness of scientometric measures. In particular, the h-frac, a fractional analogue of the h-index, is the most reliable measure across different experimental conditions.

Our analysis did not uncover unreasonable penalization of collaboration among researchers by fractional allocation measures. Fractional allocation does make explicit the expectation that each author makes a meaningful contribution to the publication’s impact. In the words of Derek de Solla Price, “Those not sharing the work, support, and responsibility do not deserve their names on the paper, even if they are the great Lord Director of the Laboratory or a titular signatory on the project. Any time you take a collaborator you must give up a share of the outcome, and you diminish your own share. That is as it should be; to do otherwise is a very cheap way of increasing apparent productivity.” [19]. Our study indicates that fractional allocation neutralizes the inflationary effects of hyperauthorship on bibliometric impact indicators, but continues to reward collaborative production of impactful scientific research [53–55].

A number of aspects of bibliometric impact indicators have not been addressed in our study. One is the normalization of bibliometric indicators across different fields, so as to enable direct comparison of scientists across fields with different publication and citation patterns [13, 14]. Another is the presence of self-citations and whether such citations should be handled differently [14, 56]. Likewise we have not addressed the role of author order and whether this order should be taken into account in automatically allocating credit for a publication’s impact [14, 57]. These are interesting avenues for future work.

Our work has both near-term and long-term implications. In the near term, our work indicates that the use of the h-index in assessing individual scientific impact should be reconsidered, and that h-frac can serve as a more robust alternative. This can ameliorate distortions introduced by contemporary authorship practices, lead to a more effective allocation of resources, and facilitate scientific discovery. In the longer term, our data, methodology, and findings can inform the science of science [11, 21] and support further quantitative analysis of research, publication, and scientific accomplishment.

## Materials and methods

### Highly-cited researchers

We construct a dataset of highly-cited researchers in four research fields: biology, computer science, economics, and physics. To begin, we retrieve a set of highly-cited researchers in each field via Google Scholar. To this end, we query Google Scholar with keywords identified by the following systematic procedure, for each field of research:

1. Begin with canonical keywords, such as “physics” for the field of physics.
2. Examine additional keywords listed by newly-retrieved highly-cited authors in their profiles. Add keywords that clearly belong to the considered field of research (e.g, “cosmology”) to the set of search queries.
3. Rerun the search with the augmented set of search queries.
4. Repeat Step 2 and 3 until convergence; i.e. until no new keywords that clearly belong to the considered field of research are identified.

The search queries resulting from this procedure can be found in S1 Fig.

The retrieved authors are sorted by the number of citations: most highly cited researchers appear first. However, the results are noisy because the queries retrieve all authors that feature the queried keyword phrases in their profiles. For example, a physicist who features “high performance computing” as a keyword phrase in their profile would be retrieved by the corresponding query. Since “high performance computing” is one of our queries for computer science researchers, the physicist would, in the absence of further validation, be added to the computer science dataset.

To clean up the initial lists compiled via Google Scholar, we cross-reference them with the Scopus database. A scientist’s Scopus profile indicates their primary research area. We use this primary research area to filter the initial lists. To this end, we need to match author profiles in Google Scholar with Scopus profiles. To perform the association, we first create a set of candidate matches by querying the Scopus database with the researcher’s name. To obtain the query name, we clean the Google Scholar profile name via simple heuristics (e.g. remove extraneous information such as links or affiliation names). To reduce false positives, we limit the candidates to Scopus profiles with more than 50 papers (more than 30 papers for economics). To perform the actual matching, we analyze the top 100 papers (sorted by citation counts) of the different candidate profiles. If we find at least three matching paper titles in the Scholar and Scopus profiles, we associate the two profiles.

After matching, we filter the authors in each field by their primary subject area in Scopus (S2 Fig). After filtering, we retain the top 1,000 authors in each field. This filtered set is derived from the top 1,186 Google Scholar profiles in biology, 1,711 in computer science, 1,632 in economics, and 1,296 in physics. This means that, in aggregate, more than two thirds of the initial Google Scholar profiles are matched to corresponding Scopus profiles with the desired primary subject area. Authors that could not be matched or do not have the requisite primary subject area are removed from the corresponding list. (They may still be retained in a list for a different field; e.g. physics rather than computer science.) One attribute of our filtering procedure is that the lists of authors in the four fields are disjoint: a scientist is only included in at most one list.

### Google Scholar data

For all 4,000 researchers, we collect their Google Scholar publications including citation data [16]. In particular, we collect (for each publication) the publication year, the number of authors, and the number of citations per year. We filter out certain publications: (i) publications that do not list authors or the publication year, (ii) patents, and (iii) duplicates marked by Google Scholar. Moreover, we noticed that the publication date and the citation years in Google Scholar are sometimes inconsistent: a publication is sometimes cited *before* is was published. As a remedy, we take the minimum of the publication year and the year of the first citation as the effective publication year.

We also noticed that Google Scholar generally under-reports the number of authors for publications with large author sets. Manual inspection indicates that Scholar does not record all authors, but only the first ∼150 authors. In particular, the maximal value of the average author count in the Scholar dataset is 230, versus 3,130 in Scopus. This is an important limitation of the Scholar data that has to be kept in mind. The consistency of our findings across the Scholar and Scopus datasets, in spite of the truncated author counts in the Scholar data, indicates that our findings are robust to such noise and bias in the data.

### Scopus data

Similar to the Google Scholar data, we collect for each of the 4,000 authors their Scopus publications with citation data [15]. Since the Scopus data is significantly less noisy than the Scholar data, no special data cleaning and filtering are required.

One salient difference between the datasets is that the Google Scholar datasets contain approximately twice as many publications and citations than the Scopus datasets. One contributing factor is that Scopus indexes only a subset of the venues crawled by Google Scholar. For example, Scopus does not index online repositories such as arXiv. In agreement with prior studies, we have found Google Scholar data to be both broader and noisier than Scopus [14]. The consistency of our findings across the Scholar and Scopus datasets highlights their robustness.

### Award data

We use awards bestowed by the scientific community as indicators of scientific reputation. To this end, we consider highly selective distinctions, some of which span multiple scientific fields, such as membership in the National Academy of Sciences, and some of which are field-specific, such as fellowship of the Econometric Society (S4A Fig, S1 Table and S1 Dataset).

Our award data collection procedure begins by compiling complete lists of laureates for each award from the respective web sites. (This is nontrivial since it requires customized parsing techniques for each award.) Next, we search these lists of laureates for names in our datasets. This search is based on the surname and the initials from each Scopus author profile in our dataset. This yields a list of candidate matches. We then manually check all candidate matches, considering the author details in the Scopus profile, such as name variations, affiliations, and subject areas, as well as details extracted from the corresponding award pages, such as bio, affiliation, and country. (S4A and S4B Fig and S1 Table).

For each laureate, we also retain the year in which the award was conferred. This is central to our measurement of correlation and predictive power over time.

## Supporting information

S1 Text Supplementary information. Contains details about the data collection, the scientometric measures, and the conducted evaluation.
(PDF)

S1 Fig Google Scholar queries used to initialize the datasets. Distribution of search queries in the initial lists of researchers; i.e. the number of researchers in the initial lists who feature the respective keyword phrase in their profile.
(PDF)

S2 Fig Scopus subject areas used for filtering the initial author list compiled from Google Scholar. The plots show the number of author profiles in the filtered datasets with the respective subject as their primary research area.
(PDF)

S3 Fig Overview of Scopus and Google Scholar datasets. Scholar (top) and Google Scholar (bottom) datasets. From left to right: Cumulative number of authors, publications, and citations per year, from 1970 onwards. Authors are considered present in the database if they have at least one publication recorded by the considered year.
(PDF)

S4 Fig Award statistics. (**A**) Cumulative number of awards indexed in our data collection. (**B**) Cumulative number of awards to scientists in our datasets. (**C**) Cumulative number of awards to scientists in each research field. (**D**) Distribution of the number of awards garnered by individual scientists.
(PDF)

S5 Fig Receiver operating characteristic (ROC) curve and area under the curve (AUC) for each research field and data source. (**A**) The horizontal axis is the accumulated fraction of scientists with no awards (*false positive rate*). The vertical axis is the fraction of awards accumulated by scientists (*true positive rate*). Larger area under the curve (AUC) indicates that a given bibliometric indicator ranks scientists who have received more awards more highly. Details are given in the text. (**B**) Numerical values of AUC for each research field and data source.
(PDF)

S6 Fig Effectiveness of scientometric measures over time for different evaluation criteria. From left to right: Kendall’s *τ*, area under the curve (AUC), Somers’ D, Goodman and Kruskal’s *γ*, Spearman’s *ρ*.
(PDF)

S7 Fig Effectiveness of scientometric measures over time for different perturbations of rankings induced by awards. From left to right: equal weight for all awards (default), higher weight for awards with < 100 laureates, binary (yes / no) award counting, random subsets of awards reveiced by researchers in our database (75% and 50%).
(PDF)

S8 Fig Effectiveness of scientometric measures over time for different subsets of researchers. From left to right: all researchers, without hyperauthors, authors with fewer citations (bottom half), authors with publication peak in [2000, 2010), authors with publication peak in [2010, 2020).
(PDF)

S1 Table Awards used in our study. The first five awards apply to all research areas (*cross*-field), while the others are field-specific (*CS* stands for *computer science*). The second-to-last column lists the total number of laureates of each award. The last column shows the number of laureates in our datasets.
(PDF)

S2 Table Effectiveness of scientometric measures. Higher is better. The most effective measure in each dataset is highlighted in bold.
(PDF)

S1 Dataset List of awards used in our study. Contains award data sources and access dates.
(CSV)

S2 Dataset Sample of hyperauthors from the Scopus physics dataset that have > 2,441 coauthors per publication on average. Contains references to Scopus profiles.
(CSV)
