# Supplementary material for: The h-index is no longer an effective correlate of scientific reputation
Source: PLoS One. 2021 Jun 28;16(6):e0253397. doi: 10.1371/journal.pone.0253397 (PMC8238192; doi:10.1371/journal.pone.0253397)
Supplement: S1 Text — Contains details about the data collection, the scientometric measures, and the conducted evaluation. (PDF) [file pone.0253397.s001.pdf]

Supplementary Information

The h-index is no longer an effective correlate of scientific reputation

Vladlen Koltun[1*], David Hafner[2]

1 Intelligent Systems Lab, Intel, Jackson, WY 83001, USA
2 Intelligent Systems Lab, Intel, 85579 Neubiberg, Germany

* vladlen.koltun@intel.com

Data Collection

Scholar Data

In total, we collect 2,624,994 (valid) publications in Google Scholar that are collectively cited 220,783,854 times. The distribution of the publications and citations among the research fields is as follows (S3 Fig(bottom)): biology accounts for 31% of the publications and 38% of the citations, computer science for 22% and 17%, economics for 12% and 10%, and physics for 35% and 35%. Our dataset offers yearly granularity from 1970 onwards.

Scopus Data

In total, the Scopus dataset comprises 1,290,219 publications with 102,405,086 citations. The distribution of publications and citations is as follows (S3 Fig(top)): biology accounts for 34% of publications and 49% of citations, computer science for 20% and 14%, economics for 6% and 4%, and physics for 41% and 33%.

Award Data

In total, we trace 1,848 distinct awards to the 4,000 scientists in our dataset. Some scientists have received multiple awards. The number of distinct scientists who have received at least one award in the dataset is 976 (24.4%). 13.3% of the researchers received exactly one award, 5.1% received two, 3.1% three, and 2.9% more than three awards (S4 Fig, D). Of the 1,848 distinct awards, 653 (35.3%) were granted to researchers in biology, 526 (28.5%) in economics, 402 (21.8%) in physics, and 267 (14.4%) in computer science (S4 Fig, C).

Scientometric Measures

The following paragraphs explain the main scientometric measures that we consider in this work.

H-Index

The h-index, originally proposed by Hirsch in 2005 [1], is defined as the maximal value of h such that h publications by the author have at least h citations each. Let N be the number of publications and let $\{c_1, \ldots, c_N\}$ the number of citations per paper in decreasing order; i.e. $c_i \geq c_j$ for $i < j$. The h-index is given by

$$\mathrm{h} = \max(h) \quad \text{s.t.} \quad c_h \geq h \,. \tag{1}$$

C-Index

We define the c-index as the total number of citations to all publications by the author:

$$\mathrm{c} = \sum_{i=1}^{N} c_i \,. \tag{2}$$

μ-Index

Lehmann et al. [2] advocated the use of the mean number of citations per paper:

$$\mu = \frac{1}{N} \sum_{i=1}^{N} c_i \,. \tag{3}$$

G-Index

Egghe's g-index [3] is a variation on the h-index. It is defined as the maximal value of g such that g publications by the author collectively have at least g^2 citations in total:

$$\mathrm{g} = \max(g) \quad \text{s.t.} \quad \sum_{i \le g} c_i \ge g^2 \,. \tag{4}$$

O-Index

The o-index, proposed by Dorogovtsev and Mendes in 2015 [4], is defined as the geometric mean of the h-index (h) and the citation count of the most-cited publication (c_1):

$$\mathrm{o} = \sqrt{\mathrm{h}\, c_1} \,. \tag{5}$$

M-Index

The m-index, proposed by Bornmann et al. [5], is defined as the median number of citations received by publications in the h-core. The h-core comprises the top h publications ranked by citation count. Thus

$$\mathrm{m} = \mathrm{median}\left(\{c_1, \ldots, c_{\mathrm{h}}\}\right) \,. \tag{6}$$

Based on these traditional scientometric measures, we define their factional counterparts (*-frac*). The fractional measures are based on citation counts $\bar{c}$ that are normalized by the number of authors per publication:

$$\bar{c} = \frac{c}{A} \,, \tag{7}$$

where A is the number of authors. The intuition is that this normalization distributes the contribution of a publication equally among the authors. This is clearly a simplification of credit allocation in science [6], but it is simple and does not introduce new parameters.

H-Frac

The fractional h-index, h-frac, is defined as

$$\text{h-frac} = \max(h) \quad \text{s.t.} \quad \bar{c}_h \ge h \,. \tag{8}$$

Here $\{\bar{c}_1, \ldots, \bar{c}_N\}$ are the normalized citation counts per paper in decreasing order; i.e. $\bar{c}_i \ge \bar{c}_j$ for $i < j$.

C-Frac

The fractional measure c-frac is the aggregate of the author's normalized citation counts:

$$\text{c-frac} = \sum_{i=1}^{N} \bar{c}_i \,. \tag{9}$$

μ-Frac

μ-frac is the mean of the normalized citation counts, averaged over all publications by the author:

$$\mu\text{-frac} = \frac{1}{N} \sum_{i=1}^{N} \bar{c}_i \,. \tag{10}$$

G-Frac

g-frac is likewise defined by analogy with the g-index using normalized citation counts:

$$\text{g-frac} = \max(g) \quad \text{s.t.} \quad \sum_{i \leq g} \bar{c}_i \geq g^2 \,. \tag{11}$$

O-Frac

We define o-frac as the geometric mean of the fractional h-index (h-frac) and the largest normalized citation count ($\bar{c}_1$):

$$\text{o-frac} = \sqrt{\text{h-frac}\; \bar{c}_1} \,. \tag{12}$$

M-Frac

The fractional counterpart of the m-index, m-frac, is the median of the normalized citation counts among the top h-frac publications ranked by normalized citation counts:

$$\text{m-frac} = \text{median}\left(\{\bar{c}_1, \ldots, \bar{c}_{\text{h-frac}}\}\right) \,. \tag{13}$$

Effectiveness of Scientometric Measures

ROC Curves

We analyze a receiver operating characteristic (ROC) curve for each dataset (S5 Fig, A). We rank the scientists by the considered scientometric measure. Lower rank corresponds to higher value of the measure. The scientist with the highest value in the dataset has rank 1. The ROC curve starts at $(0, 0)$. We iterate over the list of scientists, in order of rank r (from 1 onwards), and aggregate the awards. Step r adds the following data point to the ROC curve. The x-coordinate is the fraction of the first r scientists that have not received any award in the dataset (*false positive rate*). The y-coordinate is the fraction of the total number of awards in the dataset received by the first r scientists (*true positive rate*). By construction, the ROC curve ends, for r =1,000, at $(1, 1)$. The area under the curve (AUC) is an indicator of the effectiveness of the considered scientometric measure [7]. If a measure ranks scientists that have garnered more awards more highly, the ROC curve rises faster and the AUC is higher.

The fractional measures perform much better than their non-fractional counterparts. h-frac performs best across all research areas and datasets (S5 Fig).

In addition to the AUC, we analyze other criteria that quantify the correlation between a ranking of scientists by a certain scientometric measure and a ranking by the number of awards. If the two rankings are

similar (high correlation), the scientometric measure is taken to be a more veridical indicator of scientific reputation. We evaluate the following correlation measures.

Kendall's τ

We use the τ_b form of Kendall's τ, which accounts for ties [8]. It is defined as

$$\tau \;=\; \tau_b \;=\; \frac{C-D}{\sqrt{(C+D+T_A)\cdot(C+D+T_B)}}\,, \tag{14}$$

where C is the number of concordant and D the number of discordant pairs in two rankings A and B. T_A is the number of ties in A only and T_B is the number of ties in B only. If a tie occurs in both A and B, it is not added to either T_A or T_B. Equation (14) reduces to τ_a when no ties are present [9]:

$$\tau_a \;=\; \frac{C-D}{n\,(n-1)/2}\,, \tag{15}$$

where n is the number of elements in A or B.

Somers' D

We also measure Somers' D [10]. Somers' D of a ranking A w.r.t. a ranking B is defined as

$$\mathrm{D}_{AB} \;=\; \frac{\tau_a(A,B)}{\tau_a(B,B)}\,. \tag{16}$$

Note that Somers' D is asymmetric. In our evaluation, we set A to the ranking by the considered scientometric measure and B to the ranking based on awards.

Goodman and Kruskal's γ

Goodman and Kruskal's γ is defined as follows [11]:

$$\gamma \;=\; \frac{C-D}{C+D}\,. \tag{17}$$

Spearman's ρ

We also compute Spearman's rank correlation coefficient [12], which is defined as the Pearson correlation coefficient between the rank variables:

$$\rho \;=\; \frac{\mathrm{cov}(r_A, r_B)}{\sigma_{r_A}\,\sigma_{r_B}}\,, \tag{18}$$

where r_A and r_B are rank variables and σ_{r_A} and σ_{r_B} the corresponding standard deviations.

The results in S2 Table support the following observations. First, the fractional measures perform consistently better than their non-fractional counterparts. Furthermore, the relative order of effectiveness of scientometric measures is consistent in the different correlation statistics. This highlights the robustness of our findings. Overall, h-frac is the most effective scientometric measure in terms of correlation with scientific reputation (as indicated by scientific awards).

Of the four research fields we study, economics stands out in terms of the relative effectiveness of different scientometric measures. In economics, g-frac and o-frac appear to be the most effective measures. However, the variation between the scientometric measures in economics is substantially smaller than in the other research fields. For example, the minimal and maximal values of Kendall's τ in biology in the Scopus dataset are 0.02 and 0.34, while the minimal and maximal values for economics are 0.22 and 0.30 (S2 Table(top)). Examination of the data suggests that the field of economics has retained more classical publication patterns, with smaller author sets, fewer publications per author, and minimal hyperauthorship.

Temporal Dynamics

Effectiveness Over Time

Next, we analyze the effectiveness of scientometric measures in each year from 1990 onwards. To this end, we consider for each year Y the publication and award data up to that year. In particular, we only consider publications up to year Y as well as citations and awards up to the end of year Y. This enables us to investigate the evolution of the effectiveness of scientometric measures over time (Fig. 2(top)).

We again observe that the fractional measures perform better than their non-fractional counterparts. While most measures tend to decrease in effectiveness over time, the factional measures are more stable. The difference between the fractional and non-fractional measures increases over time. From 2014 onwards, *all* fractional measures are on average more effective than any of the traditional measures (Fig. 2A(top)). Among all measures, h-frac is the most effective in terms of correlation with scientific reputation (Fig. 2(top)).

Predictive Power Over Time

We also investigate the temporal evolution of the predictive power of scientometric measures. Our aim is to quantify how well a scientometric measure predicts *future* scientific reputation. To this end, we compare a ranking of scientists induced by the considered scientometric measure in year Y to a ranking induced by awards garnered up to year Y + X. A high correlation among these two rankings implies that the scientometric measure is a good predictor of scientific reputation X years into the future. We compute the same correlation measures defined earlier and take $X = 5$ as our default. That is, we measure the ability of scientometric indicators to predict scientific reputation (as evidenced by awards) 5 years in advance (Fig. 2(bottom)).

Our findings on predictive power are consistent with our earlier findings: The fractional measures are consistently more predictive than their non-fractional counterparts. All scientometric measures tend to decline in predictive power over time, but the fractional measures are more stable. The differences between fractional and non-fractional measures increase over time. From 2014 onwards, all fractional measures are more predictive than the traditional ones (Fig. 2A(bottom)). h-frac is the most predictive scientometric measure (Fig. 2(bottom)).

References

1. Hirsch JE. An index to quantify an individual's scientific research output. Proceedings of the National Academy of Sciences. 2005;102(46):16569–16572.

2. Lehmann S, Jackson AD, Lautrup BE. Measures for measures. Nature. 2006;444(7122):1003–1004.

3. Egghe L. Theory and practise of the g-index. Scientometrics. 2006;69(1):131–152.

4. Dorogovtsev SN, Mendes JFF. Ranking scientists. Nature Physics. 2015;11(11):882–883.

5. Bornmann L, Mutz R, Daniel HD. Are there better indices for evaluation purposes than the h index? A comparison of nine different variants of the h index using data from biomedicine. Journal of the American Society for Information Science and Technology. 2008;59(5):830–837.

6. Waltman L. A review of the literature on citation impact indicators. Journal of Informetrics. 2016;10(2):365–391.

7. Sinatra R, Wang D, Deville P, Song C, Barabási AL. Quantifying the evolution of individual scientific impact. Science. 2016;354(6312).

8. Kendall MG. The treatment of ties in ranking problems. Biometrika. 1945;33(3):239–251.

9. Kendall MG. A new measure of rank correlation. Biometrika. 1938;30(1/2):81–93.

10. Somers RH. A new asymmetric measure of association for ordinal variables. American Sociological Review. 1962;27(6):799–811.

11. Goodman LA, Kruskal WH. Measures of association for cross classifications. Journal of the American Statistical Association. 1954;49(268):732–764.

12. Lovie AD. Who discovered Spearman's rank correlation? British Journal of Mathematical and Statistical Psychology. 1995;48(2):255–269.
