# Supplementary material for: The h-index is no longer an effective correlate of scientific reputation
Source: PLoS One. 2021 Jun 28;16(6):e0253397. doi: 10.1371/journal.pone.0253397 (PMC8238192; doi:10.1371/journal.pone.0253397)
Supplement: S1 Fig — Distribution of search queries in the initial lists of researchers; i.e. the number of researchers in the initial lists who feature the respective keyword phrase in their profile. (PDF) [file pone.0253397.s002.pdf]

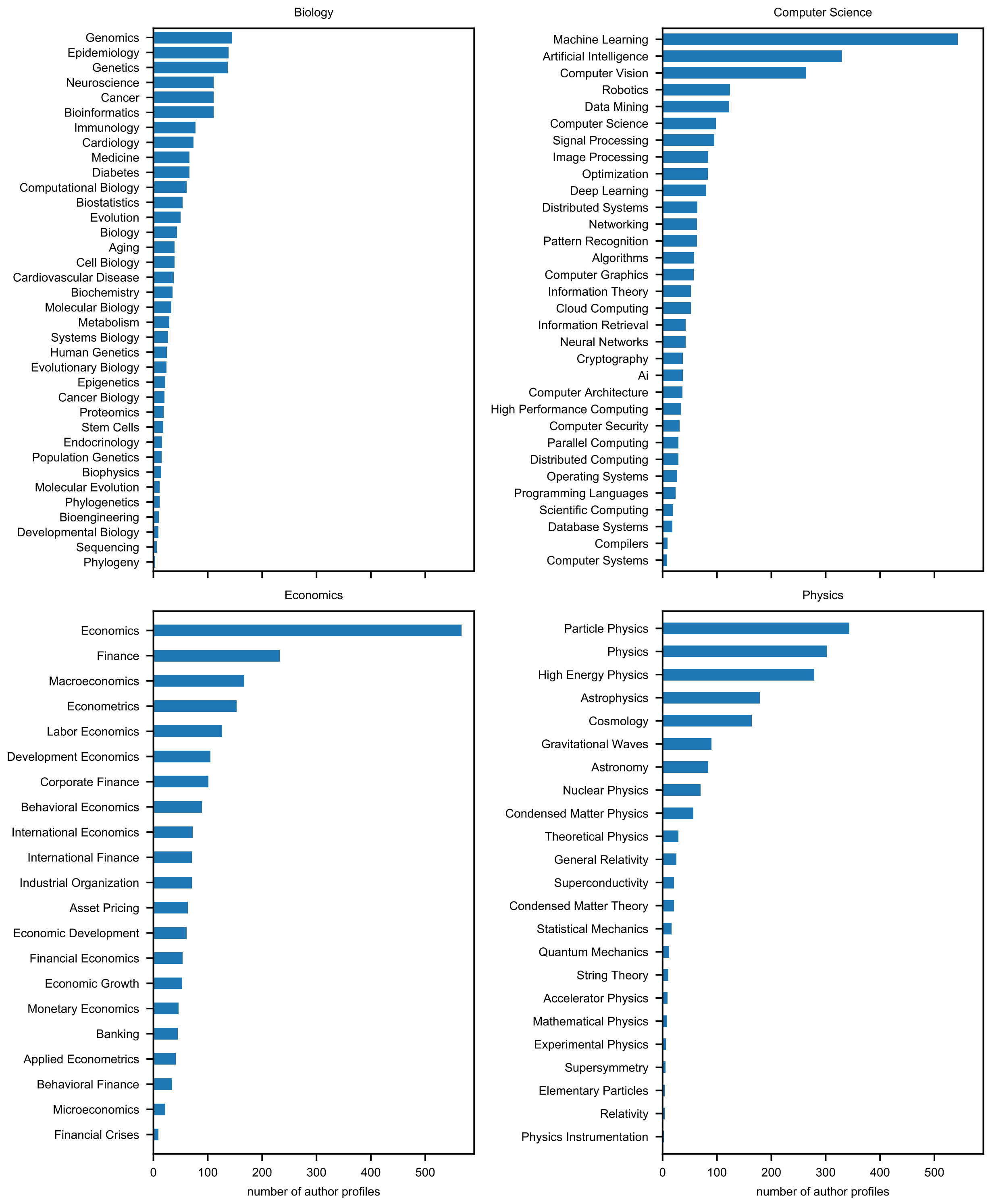

S1 Fig. Google Scholar queries used to initialize the datasets. Distribution of search queries in the initial lists of researchers; i.e. the number of researchers in the initial lists who feature the respective keyword phrase in their profile.
