# Supplementary material for: The h-index is no longer an effective correlate of scientific reputation
Source: PLoS One. 2021 Jun 28;16(6):e0253397. doi: 10.1371/journal.pone.0253397 (PMC8238192; doi:10.1371/journal.pone.0253397)
Supplement: S2 Fig — The plots show the number of author profiles in the filtered datasets with the respective subject as their primary research area. (PDF) [file pone.0253397.s003.pdf]

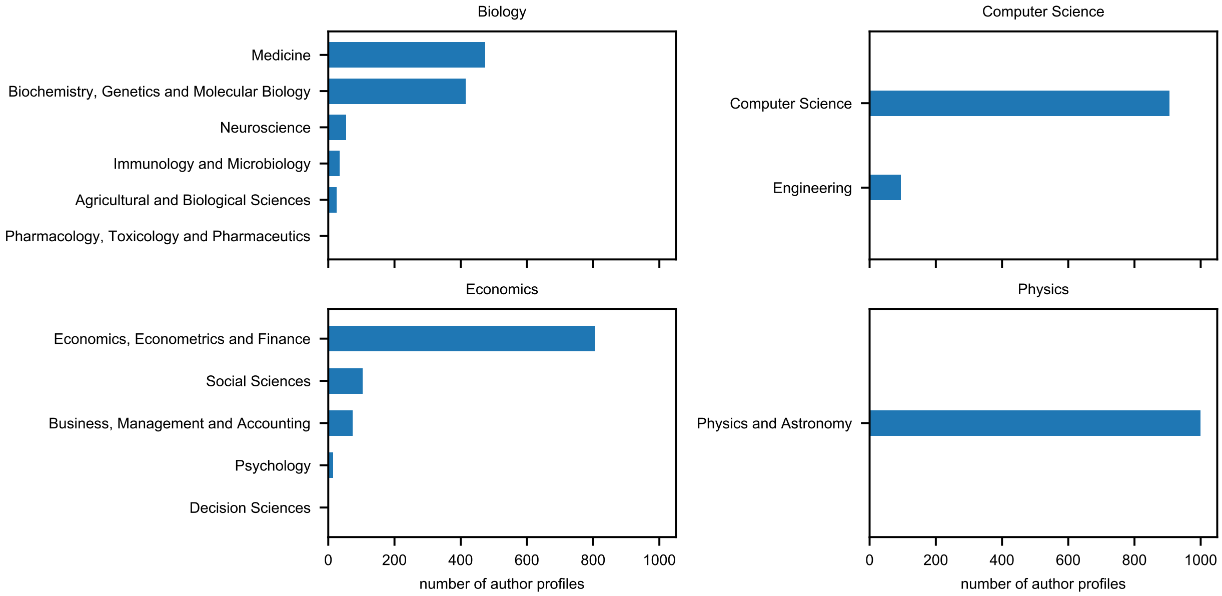

S2 Fig. Scopus subject areas used for filtering the initial author list compiled from Google Scholar. The plots show the number of author profiles in the filtered datasets with the respective subject as their primary research area.
