# Supplementary material for: The h-index is no longer an effective correlate of scientific reputation
Source: PLoS One. 2021 Jun 28;16(6):e0253397. doi: 10.1371/journal.pone.0253397 (PMC8238192; doi:10.1371/journal.pone.0253397)
Supplement: S3 Fig — Scholar (top) and Google Scholar (bottom) datasets. From left to right: Cumulative number of authors, publications, and citations per year, from 1970 onwards. Authors are considered present in the database if they have at least one publication recorded by the considered year. (PDF) [file pone.0253397.s004.pdf]

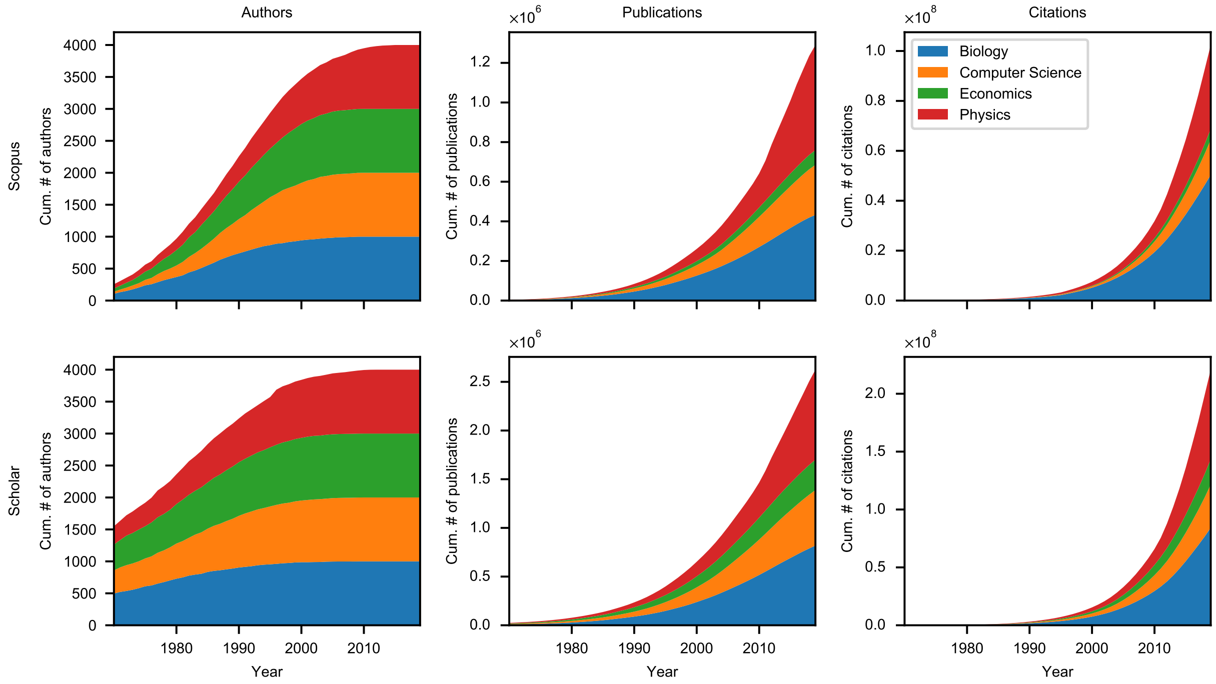

S3 Fig. Overview of Scopus and Google Scholar datasets. Scholar (top) and Google Scholar (bottom) datasets. From left to right: Cumulative number of authors, publications, and citations per year, from 1970 onwards. Authors are considered present in the database if they have at least one publication recorded by the considered year.
