# Supplementary material for: The h-index is no longer an effective correlate of scientific reputation
Source: PLoS One. 2021 Jun 28;16(6):e0253397. doi: 10.1371/journal.pone.0253397 (PMC8238192; doi:10.1371/journal.pone.0253397)
Supplement: S4 Fig — (A) Cumulative number of awards indexed in our data collection. (B) Cumulative number of awards to scientists in our datasets. (C) Cumulative number of awards to scientists in each research field. (D) Distribution of the number of awards garnered by individual scientists. (PDF) [file pone.0253397.s005.pdf]

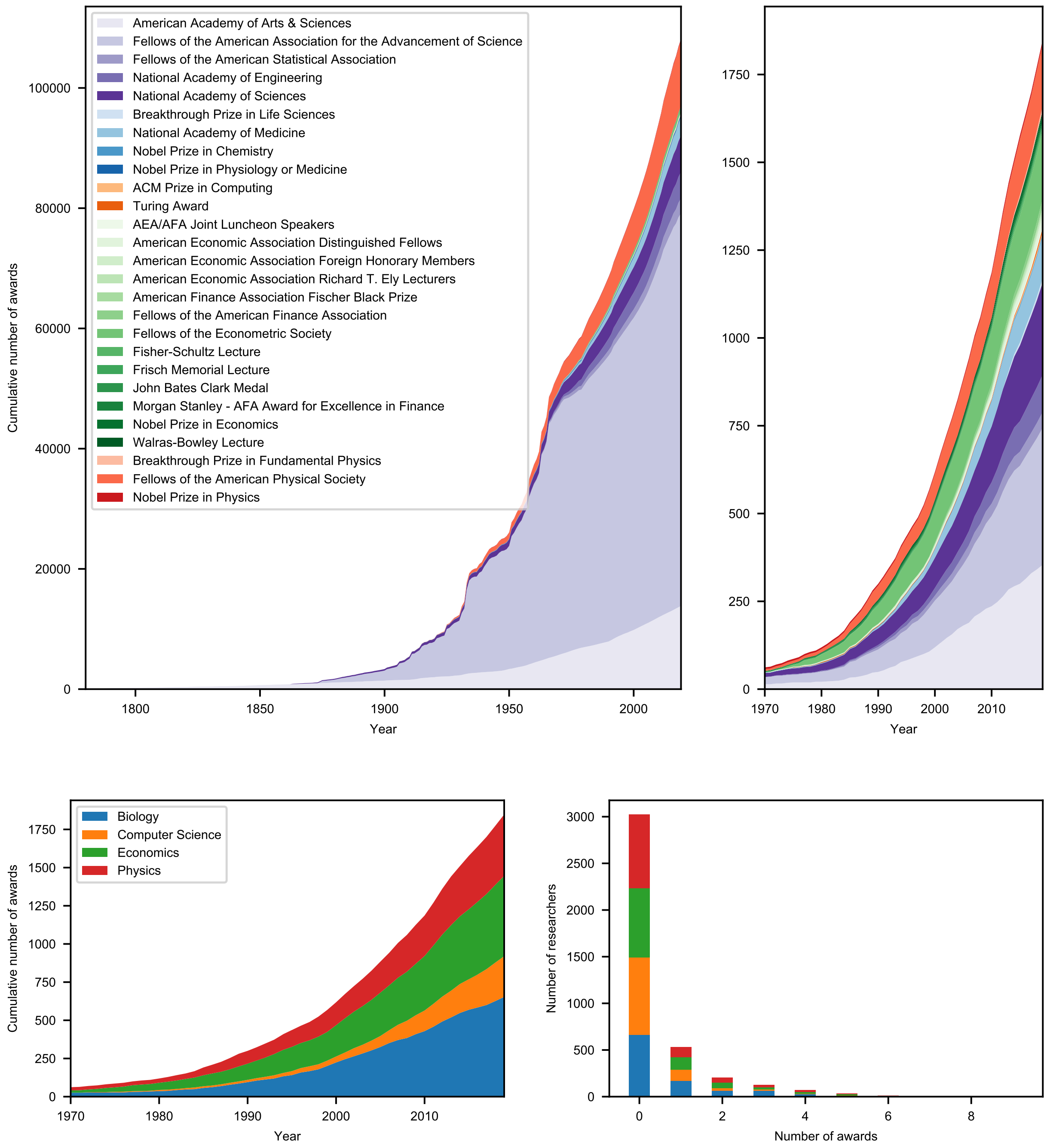

S4 Fig. Award statistics. (**A**) Cumulative number of awards indexed in our data collection. (**B**) Cumulative number of awards to scientists in our datasets. (**C**) Cumulative number of awards to scientists in each research field. (**D**) Distribution of the number of awards garnered by individual scientists.
