# Supplementary material for: The h-index is no longer an effective correlate of scientific reputation
Source: PLoS One. 2021 Jun 28;16(6):e0253397. doi: 10.1371/journal.pone.0253397 (PMC8238192; doi:10.1371/journal.pone.0253397)
Supplement: S5 Fig — (A) The horizontal axis is the accumulated fraction of scientists with no awards (false positive rate). The vertical axis is the fraction of awards accumulated by scientists (true positive rate). Larger area under the curve (AUC) indicates that a given bibliometric indicator ranks scientists who have received more awards more highly. Details are given in the text. (B) Numerical values of AUC for each research field and data source. (PDF) [file pone.0253397.s006.pdf]

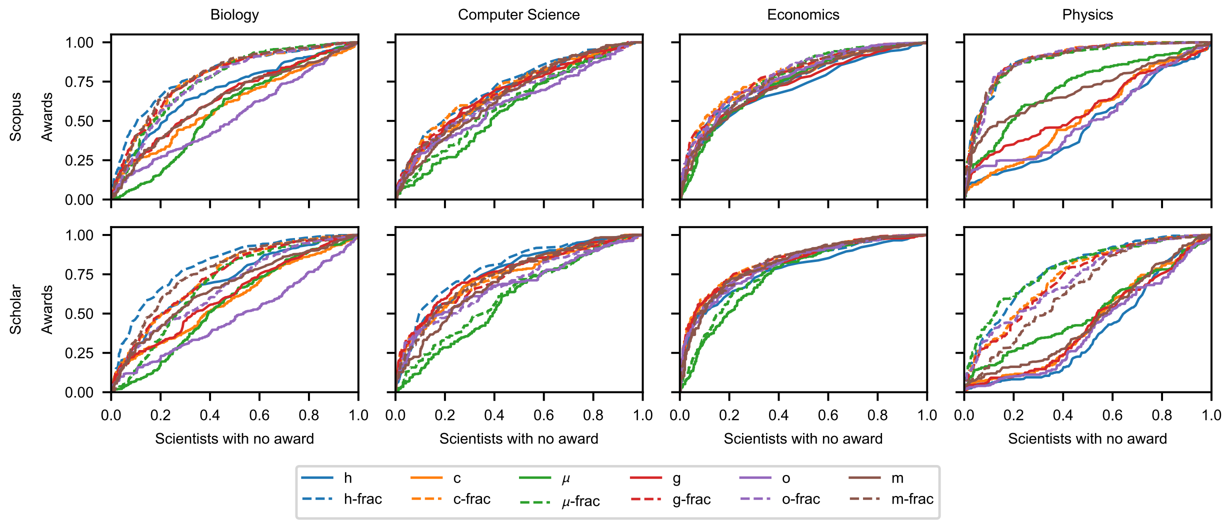

	Scopus				Scholar				
Measure	Bio	CS	Eco	Phy	Bio	CS	Eco	Phy	Avg.
c	0.60	0.68	0.77	0.54	0.59	0.74	0.83	0.45	0.65
μ	0.57	0.59	0.74	0.71	0.56	0.59	0.75	0.51	0.63
h	0.69	0.69	0.71	0.49	0.70	0.76	0.78	0.39	0.65
g	0.65	0.69	0.73	0.58	0.61	0.76	0.82	0.44	0.66
m	0.63	0.66	0.74	0.69	0.66	0.71	0.82	0.47	0.67
o	0.53	0.62	0.77	0.52	0.48	0.68	0.81	0.41	0.60
c-frac	0.77	0.71	0.79	**0.90**	0.74	0.74	0.83	0.74	0.78
μ-frac	0.76	0.62	0.77	0.88	0.68	0.62	0.77	**0.79**	0.74
h-frac	**0.80**	**0.72**	0.76	0.89	**0.81**	**0.79**	0.81	0.78	**0.80**
g-frac	0.78	0.69	0.79	0.89	0.73	0.74	**0.84**	0.73	0.77
m-frac	0.78	0.69	0.77	0.89	0.76	0.74	0.82	0.66	0.77
o-frac	0.75	0.66	**0.79**	0.89	0.67	0.69	0.82	0.72	0.75

S5 Fig. Receiver operating characteristic (ROC) curve and area under the curve (AUC) for each research field and data source. (**A**) The horizontal axis is the accumulated fraction of scientists with no awards (*false positive rate*). The vertical axis is the fraction of awards accumulated by scientists (*true positive rate*). Larger area under the curve (AUC) indicates that a given bibliometric indicator ranks scientists who have received more awards more highly. Details are given in the text. (**B**) Numerical values of AUC for each research field and data source
