# Supplementary material for: The h-index is no longer an effective correlate of scientific reputation
Source: PLoS One. 2021 Jun 28;16(6):e0253397. doi: 10.1371/journal.pone.0253397 (PMC8238192; doi:10.1371/journal.pone.0253397)
Supplement: S6 Fig — From left to right: Kendall’s τ, area under the curve (AUC), Somers’ D, Goodman and Kruskal’s γ, Spearman’s ρ. (PDF) [file pone.0253397.s007.pdf]

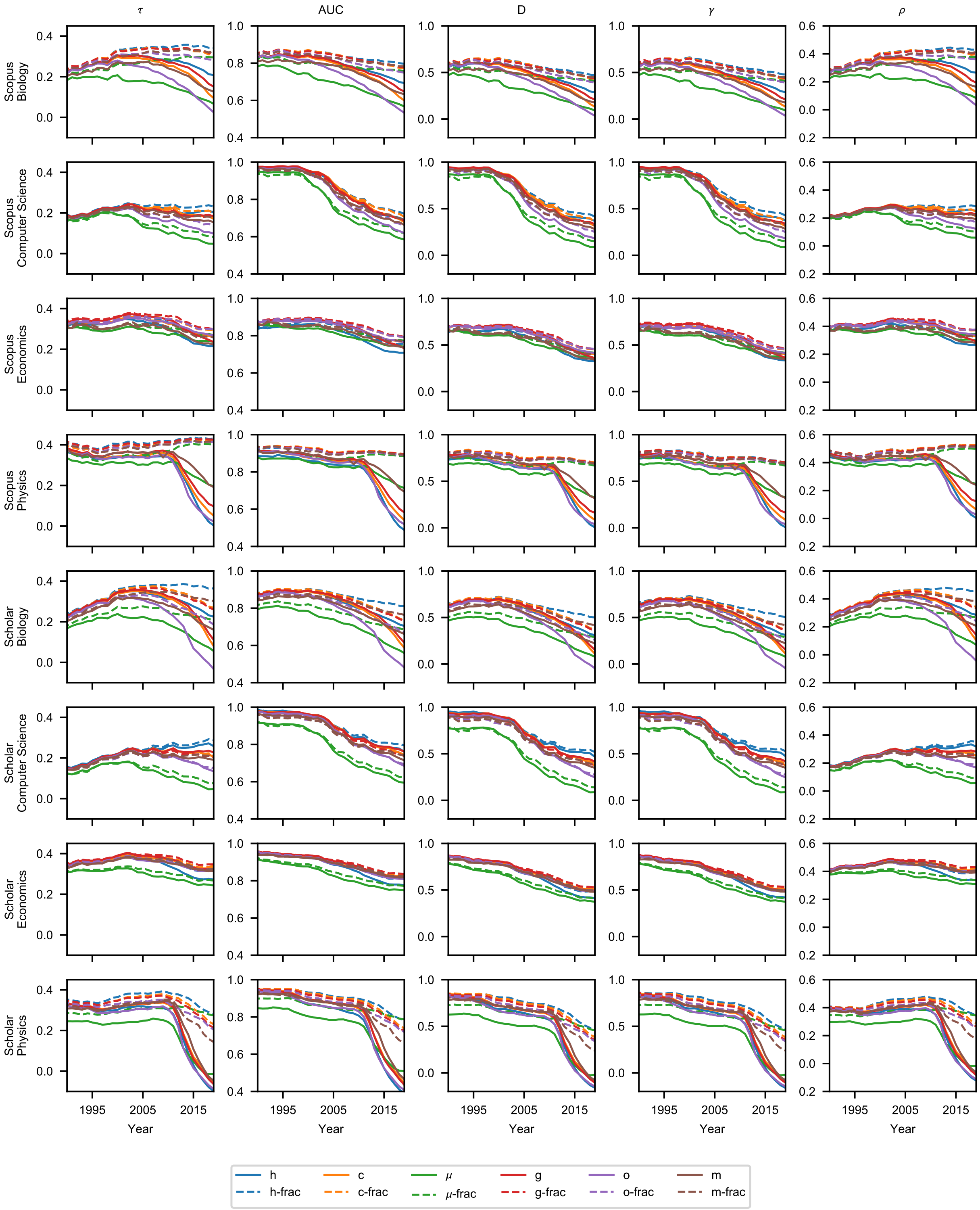

S6 Fig. Effectiveness of scientometric measures over time for different evaluation criteria. From left to right: Kendall's τ, area under the curve (AUC), Somers' D, Goodman and Kruskal's γ, Spearman's ρ.
