# Supplementary material for: The h-index is no longer an effective correlate of scientific reputation
Source: PLoS One. 2021 Jun 28;16(6):e0253397. doi: 10.1371/journal.pone.0253397 (PMC8238192; doi:10.1371/journal.pone.0253397)
Supplement: S7 Fig — From left to right: equal weight for all awards (default), higher weight for awards with < 100 laureates, binary (yes / no) award counting, random subsets of awards reveiced by researchers in our database (75% and 50%). (PDF) [file pone.0253397.s008.pdf]

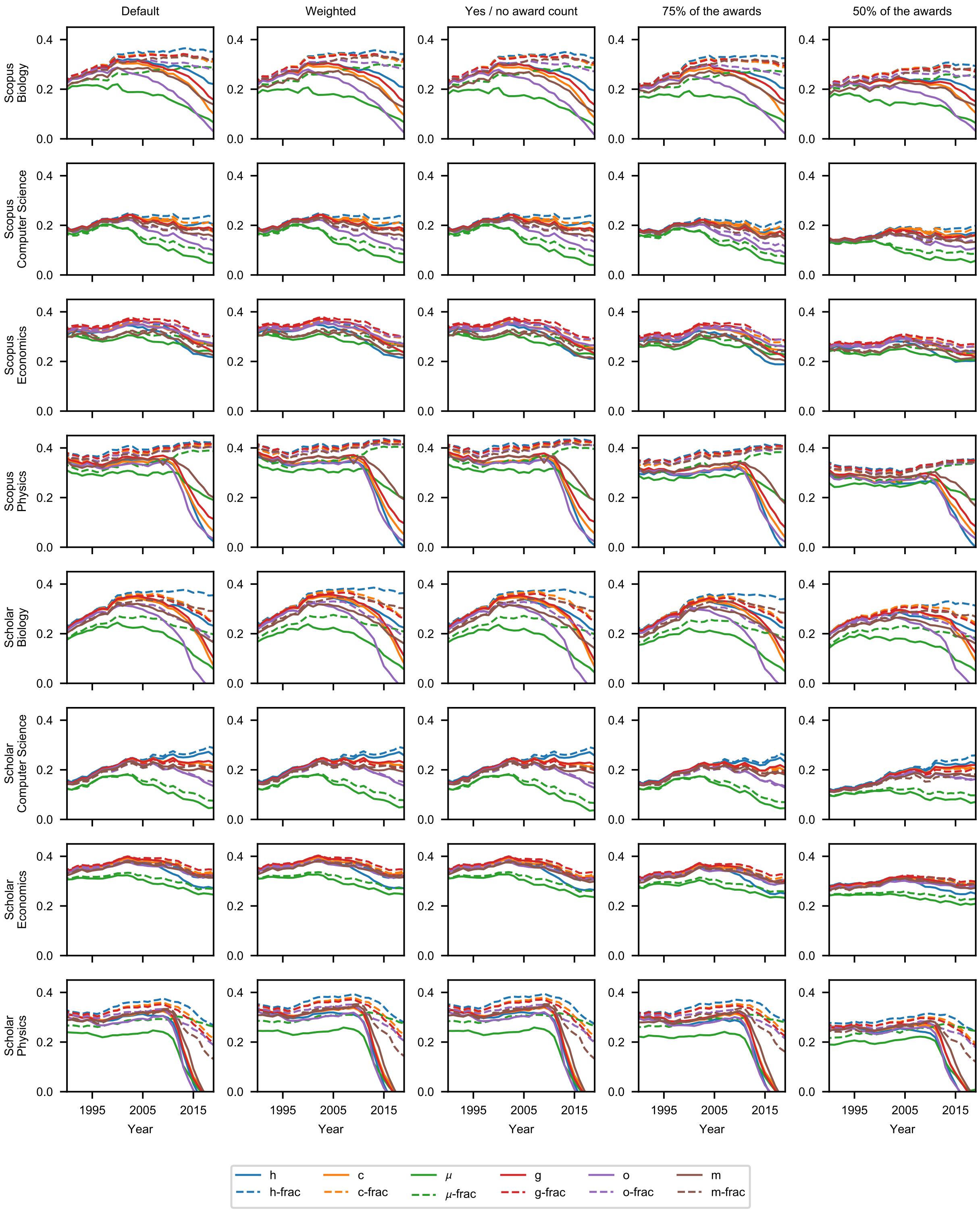

S7 Fig. Effectiveness of scientometric measures over time for different perturbations of rankings induced by awards. From left to right: equal weight for all awards (default), higher weight for awards with < 100 laureates, binary (yes / no) award counting, random subsets of awards reveiced by researchers in our database (75% and 50%).
