# Supplementary material for: The h-index is no longer an effective correlate of scientific reputation
Source: PLoS One. 2021 Jun 28;16(6):e0253397. doi: 10.1371/journal.pone.0253397 (PMC8238192; doi:10.1371/journal.pone.0253397)
Supplement: S8 Fig — From left to right: all researchers, without hyperauthors, authors with fewer citations (bottom half), authors with publication peak in [2000, 2010), authors with publication peak in [2010, 2020). (PDF) [file pone.0253397.s009.pdf]

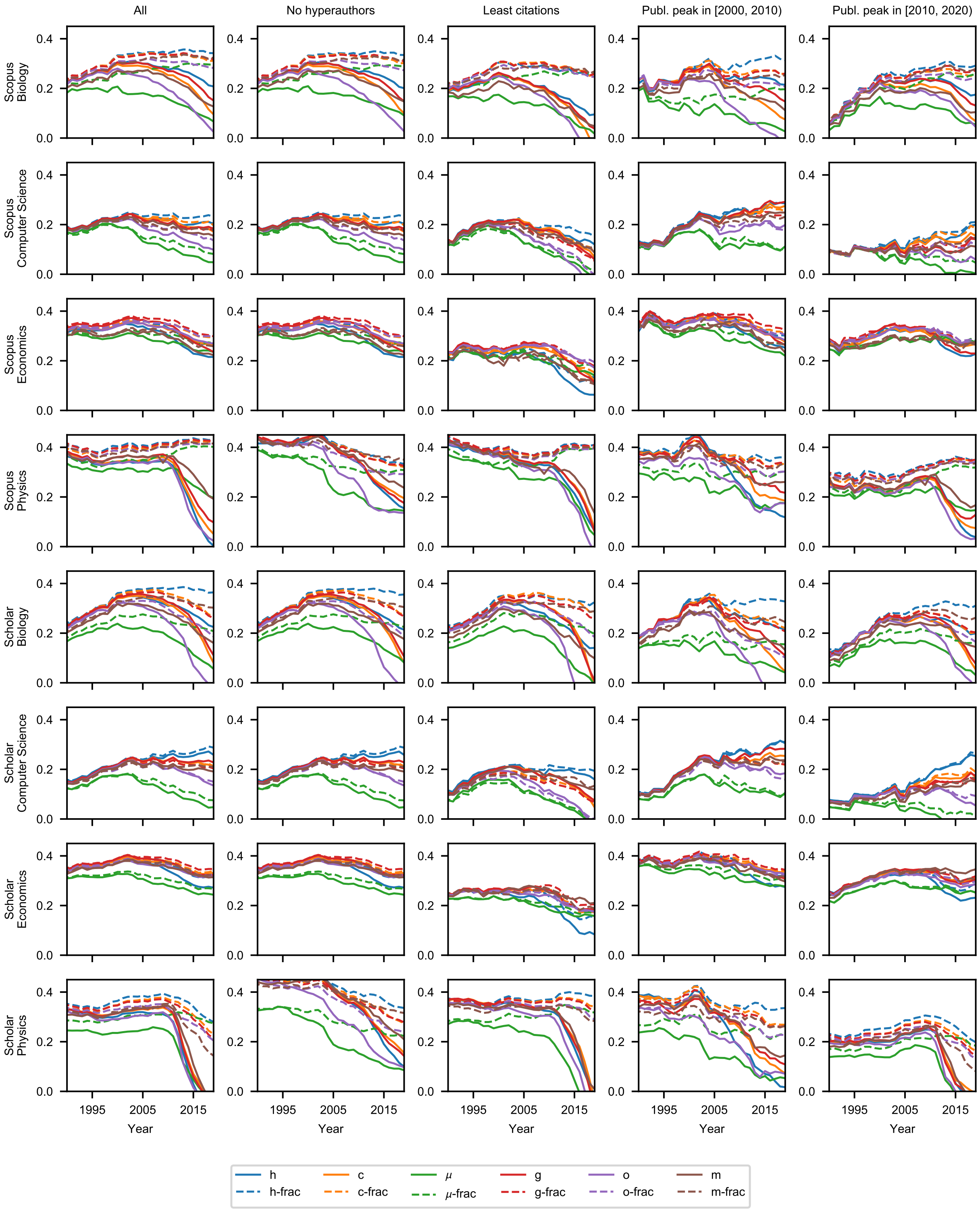

S8 Fig. Effectiveness of scientometric measures over time for different subsets of researchers. From left to right: all researchers, without hyperauthors, authors with fewer citations (bottom half), authors with publication peak in $[2000, 2010)$, authors with publication peak in $[2010, 2020)$.
