# Supplementary material for: The h-index is no longer an effective correlate of scientific reputation
Source: PLoS One. 2021 Jun 28;16(6):e0253397. doi: 10.1371/journal.pone.0253397 (PMC8238192; doi:10.1371/journal.pone.0253397)
Supplement: S1 Table — The first five awards apply to all research areas (cross-field), while the others are field-specific (CS stands for computer science). The second-to-last column lists the total number of laureates of each award. The last column shows the number of laureates in our datasets. (PDF) [file pone.0253397.s010.pdf]

S1 Table. Awards used in our study. The first five awards apply to all research areas (*cross*-field), while the others are field-specific (*CS* stands for *computer science*). The second-to-last column lists the total number of laureates of each award. The last column shows the number of laureates in our datasets.

	Award	Laureates	Matches
Cross	American Academy of Arts & Sciences	13,837	354
	Fellows of the American Association for the Advancement of Science	65,303	390
	Fellows of the American Statistical Association	2,485	45
	National Academy of Engineering	4,401	106
	National Academy of Sciences	6,085	263
Biology	Breakthrough Prize in Life Sciences	48	11
	National Academy of Medicine	2,980	121
	Nobel Prize in Chemistry	184	5
	Nobel Prize in Physiology or Medicine	219	2
CS	ACM Prize in Computing	13	7
	Turing Award	70	9
Economics	AEA/AFA Joint Luncheon Speakers	58	10
	American Economic Association Distinguished Fellows	172	25
	American Economic Association Foreign Honorary Members	40	11
	American Economic Association Richard T. Ely Lecturers	58	14
	American Finance Association Fischer Black Prize	8	4
	Fellows of the American Finance Association	66	22
	Fellows of the Econometric Society	719	194
	Fisher-Schultz Lecture	54	13
	Frisch Memorial Lecture	9	2
	John Bates Clark Medal	42	13
	Morgan Stanley - AFA Award for Excellence in Finance	5	0
	Nobel Prize in Economics	84	17
	Walras-Bowley Lecture	48	12
Physics	Breakthrough Prize in Fundamental Physics	33	13
	Fellows of the American Physical Society	10,902	178
	Nobel Prize in Physics	213	10
