# Supplementary material for: The h-index is no longer an effective correlate of scientific reputation
Source: PLoS One. 2021 Jun 28;16(6):e0253397. doi: 10.1371/journal.pone.0253397 (PMC8238192; doi:10.1371/journal.pone.0253397)
Supplement: S2 Table — Higher is better. The most effective measure in each dataset is highlighted in bold. (PDF) [file pone.0253397.s011.pdf]

S2 Table. Effectiveness of scientometric measures. Higher is better. The most effective measure in each dataset is highlighted in bold.

		Scopus				Scholar				
	Measure	Bio	CS	Eco	Phy	Bio	CS	Eco	Phy	Avg.
Kendall's τ	c	0.10	0.17	0.26	0.05	0.08	0.20	0.33	-0.05	0.14
	μ	0.07	0.05	0.24	0.20	0.06	0.05	0.24	-0.01	0.11
	h	0.21	0.20	0.22	0.00	0.22	0.26	0.27	-0.10	0.16
	g	0.15	0.18	0.24	0.10	0.11	0.23	0.32	-0.06	0.16
	m	0.13	0.15	0.22	0.19	0.16	0.19	0.31	-0.05	0.16
	o	0.02	0.10	0.27	0.02	-0.03	0.13	0.32	-0.09	0.09
	c-frac	0.31	0.20	0.29	0.42	0.27	0.21	0.34	0.23	0.28
	μ-frac	0.30	0.08	0.27	0.40	0.21	0.08	0.27	0.27	0.23
	h-frac	**0.34**	**0.23**	0.27	**0.43**	**0.36**	**0.29**	0.32	**0.28**	**0.32**
	g-frac	0.32	0.18	0.30	0.43	0.26	0.20	**0.35**	0.21	0.28
	m-frac	0.32	0.18	0.26	0.41	0.30	0.21	0.32	0.14	0.27
	o-frac	0.28	0.14	**0.30**	0.41	0.19	0.15	0.33	0.20	0.25
Somers' D	c	0.13	0.31	0.40	0.08	0.11	0.37	0.51	-0.09	0.23
	μ	0.09	0.09	0.36	0.33	0.08	0.09	0.37	-0.02	0.17
	h	0.29	0.37	0.32	0.01	0.31	0.47	0.41	-0.16	0.25
	g	0.21	0.33	0.36	0.16	0.15	0.41	0.49	-0.11	0.25
	m	0.18	0.28	0.34	0.32	0.22	0.35	0.48	-0.08	0.26
	o	0.03	0.18	0.41	0.04	-0.05	0.25	0.49	-0.15	0.15
	c-frac	0.43	0.36	0.45	**0.70**	0.37	0.39	0.52	0.38	0.45
	μ-frac	0.41	0.15	0.41	0.67	0.29	0.14	0.41	0.46	0.37
	h-frac	**0.47**	**0.42**	0.40	0.69	**0.50**	**0.52**	0.49	**0.46**	**0.49**
	g-frac	0.44	0.32	0.45	0.69	0.36	0.37	**0.53**	0.35	0.44
	m-frac	0.45	0.33	0.40	0.69	0.42	0.39	0.50	0.24	0.42
	o-frac	0.39	0.25	**0.46**	0.69	0.26	0.27	0.50	0.34	0.39
Goodman and Kruskal's γ	c	0.13	0.31	0.40	0.08	0.11	0.37	0.51	-0.09	0.23
	μ	0.09	0.09	0.36	0.33	0.08	0.09	0.37	-0.02	0.17
	h	0.29	0.37	0.34	0.01	0.31	0.47	0.42	-0.16	0.26
	g	0.21	0.34	0.36	0.16	0.15	0.42	0.50	-0.11	0.25
	m	0.18	0.28	0.34	0.32	0.22	0.35	0.48	-0.08	0.26
	o	0.03	0.18	0.41	0.04	-0.05	0.25	0.49	-0.15	0.15
	c-frac	0.43	0.36	0.45	0.70	0.37	0.39	0.52	0.38	0.45
	μ-frac	0.41	0.15	0.41	0.67	0.29	0.14	0.41	0.46	0.37
	h-frac	**0.48**	**0.43**	0.42	**0.70**	**0.51**	**0.53**	0.50	**0.47**	**0.50**
	g-frac	0.44	0.32	**0.46**	0.70	0.36	0.37	**0.53**	0.35	0.44
	m-frac	0.45	0.33	0.40	0.69	0.42	0.39	0.50	0.24	0.42
	o-frac	0.39	0.25	0.46	0.69	0.26	0.27	0.50	0.34	0.39
Spearman's ρ	c	0.12	0.21	0.33	0.06	0.10	0.25	0.41	-0.07	0.18
	μ	0.08	0.06	0.30	0.25	0.07	0.06	0.31	-0.02	0.14
	h	0.26	0.25	0.27	0.00	0.29	0.32	0.34	-0.12	0.20
	g	0.19	0.23	0.29	0.12	0.14	0.28	0.40	-0.08	0.20
	m	0.16	0.19	0.28	0.24	0.21	0.24	0.39	-0.06	0.21
	o	0.03	0.12	0.34	0.03	-0.04	0.17	0.40	-0.11	0.12
	c-frac	0.40	0.24	0.37	**0.53**	0.34	0.26	0.42	0.29	0.36
	μ-frac	0.38	0.10	0.34	0.50	0.27	0.09	0.34	0.34	0.30
	h-frac	**0.43**	**0.28**	0.33	0.52	**0.46**	**0.35**	0.40	**0.35**	**0.39**
	g-frac	0.40	0.22	0.37	0.52	0.33	0.25	**0.43**	0.27	0.35
	m-frac	0.41	0.22	0.33	0.51	0.38	0.26	0.41	0.18	0.34
	o-frac	0.36	0.17	**0.38**	0.51	0.24	0.19	0.41	0.26	0.31
